# New Insights into Different Reproductive Effort and Sexual Recruitment Contribution between Two Geographic *Zostera marina* L. Populations in Temperate China

**DOI:** 10.3389/fpls.2018.00015

**Published:** 2018-02-12

**Authors:** Shaochun Xu, Pengmei Wang, Yi Zhou, Xiaomei Zhang, Ruiting Gu, Xujia Liu, Bingjian Liu, Xiaoyue Song, Shuai Xu, Shidong Yue

**Affiliations:** ^1^CAS Key Laboratory of Marine Ecology and Environmental Sciences, Institute of Oceanology, Chinese Academy of Sciences, Qingdao, China; ^2^Laboratory for Marine Ecology and Environmental Science, Qingdao National Laboratory for Marine Science and Technology, Qingdao, China; ^3^College of Earth Sciences, University of Chinese Academy of Sciences, Beijing, China

**Keywords:** *Zostera marina* L., sexual reproduction, clonal growth, seed bank, seedling, flowering shoot, population recruitment, temperature regime

## Abstract

Seagrasses are important components of global coastal ecosystems, and the eelgrass *Zostera marina* L. is widely distributed along the Atlantic and Pacific coasts in the temperate northern hemisphere, but limited datum related to the contribution of sexual reproduction to population recruitment have been reported. This study aimed to understand eelgrass sexual reproduction and population recruitment in Swan Lake (SLL), and Huiquan Bay (HQB) was included for comparison. Random sampling, permanent quadrats or cores and laboratory seed germination-based experimental methods were employed. The flowering, seed production, seed banks, seed germination, seedling survival, and seedling growth of eelgrass were investigated from July 2014 to December 2015 to evaluate the contribution of sexual reproduction to population recruitment. Results indicated a dominant role of asexual reproduction in HQB, while sexual reproduction played a relatively important role in SLL. The highest flowering shoot density in SLL was 517.27 ± 504.29 shoots m^−2^ (June) and represented 53.34% of the total shoots at the center site. The potential seed output per reproductive shoot and per unit area in SLL were 103.67 ± 37.95 seeds shoot^−1^ and 53,623.66 ± 19,628.11 seeds m^−2^, respectively. The maximum seed bank density in SLL was 552.21 ± 204.94 seeds m^−2^ (October). Seed germination mainly occurred from the middle of March to the end of May, and the highest seedling density was 296.88 ± 274.27 seedlings m^−2^ in April. The recruitment from seedlings accounted for 41.36% of the *Z. marina* population recruitment at the center site, while the sexual recruitment contribution at the patch site (50.52%) was greater than that at the center site. Seeds in SLL were acclimated to spring germination, while in HQB, they were acclimated to autumn germination (early October–late November). Seed bank density in HQB was very low, with a value of 254.35 ± 613.34 seeds m^−2^ (early October). However, seeds in HQB were significantly larger and heavier than those in SLL (size: *P* = 0.004; weight: *P* < 0.001). The recruitment from seedlings accounted for as low as 2.53% of the *Z. marina* population recruitment in HQB. Our laboratory seed germination experiment, which was conducted in autumn, showed that the seed germination percent in HQB was significantly greater than in SLL at optimal germination temperatures (10 and 15°C; *P* < 0.001). A laboratory seed germination test at suitable temperature may be a potential novel approach to identify the ecological differences among different geographic populations. It is suggested that the *Z. marina* population recruitment may have different strategies and adapt to specific local conditions, such as in SLL and HQB, and the temperature regime may control morphological and phonological variations.

## Introduction

Seagrasses, submerged marine angiosperms, are widely distributed along the tropical and temperate coastlines of the world. Seagrass communities provide habitats, foods, and nurseries for a variety of marine organisms (Costanza et al., 1997; Beck et al., 2001; Jackson et al., 2001; Duffy, 2006; Heck and Valentine, 2006; Verweij et al., 2008; Barbier et al., 2011; Liu et al., 2013; Tol et al., 2016; Taylor et al., 2017), increase water clarity by enhancing sedimentation and remove nutrients from the water column by regulating nutrient cycles (Barbier et al., 2011), and function as key sites for global carbon storage in the biosphere (Fourqurean et al., 2012; Trevathan-Tackett et al., 2017). However, seagrass meadows are disappearing at an alarming rate worldwide (Short et al., 2011) because of anthropogenic activities, such as port infrastructure development and dredging (Erftemeijer and Robin Lewis, 2006; Orth et al., 2006a; Waycott et al., 2009; Unsworth et al., 2017). These issues have led to a global seagrass conservation movement and the promotion of seagrass resilience to mitigate seagrass losses and to enhance critical ecosystem functions (Orth et al., 2006a; Shafer and Bergstrom, 2010; Unsworth and Cullen, 2010; Cunha et al., 2014; Zhou et al., 2014; Unsworth et al., 2015; Suykerbuyk et al., 2016). To improve seagrass resilience in the face of rapid and global environmental change (e.g., global warming), studying seagrass population recruitment is necessary because it plays a critical role in improving seagrass resilience to global warming through genetic diversity (Orth et al., 2000, 2012; Ehlers et al., 2008; Hughes and Stachowicz, 2009; Jarvis and Moore, 2010; Tanner and Parham, 2010).

Seagrasses colonize the sea both asexually, through clonal growth, and sexually, through the production of flowers and seeds (Phillips and Meñez, 1988; Darnell et al., 2015). Asexual reproduction is the main contributor to the population recruitment of seagrass meadows (Duarte and Sandjensen, 1990; Procaccini and Mazzella, 1998; Hemminga and Duarte, 2000; Rasheed, 2004), while successful population recruitment from sexual reproduction, which is constrained by bottlenecks along the reproductive cycle of seagrasses (e.g., flowering, fruiting, seed germination and seedling survival; Hemminga and Duarte, 2000; Orth et al., 2006b) is extremely low (Hemminga and Duarte, 2000; Cabaco and Santos, 2009). However, sexual reproduction is the only way to maintain the population's genetic diversity (Ackerman, 2006; Reynolds et al., 2012, 2013), which improves the ability of seagrasses to withstand adverse environments and recover from disturbance more rapidly (Coyer et al., 2004; Hughes and Stachowicz, 2004, 2009; Ehlers et al., 2008; Cabaco and Santos, 2012). It is also important for colonizing new habitats and in recolonizing areas of large-scale decline or complete destruction by dispersing seeds from parental meadows (Howe and Smallwood, 1982; Thayer et al., 1984; Marba and Walker, 1999; Harwell and Orth, 2002; Plus et al., 2003; Rasheed, 2004; Greve et al., 2005; Lee et al., 2007; Kallstrom et al., 2008; Jarvis and Moore, 2010; Kendrick et al., 2012). These results indicate the increasing importance of seeds in seagrass conservation and restoration and have led to recent studies on sexual recruitment (Orth et al., 2000, 2012; Jarvis and Moore, 2010; Tanner and Parham, 2010; Furman et al., 2015; Balestri et al., 2017).

Eelgrass (*Zostera marina*) is the most widely distributed species of seagrass throughout the Atlantic and Pacific coasts of the temperate northern hemisphere (Hartog, 1972; Olesen, 1999; Green and Short, 2003; Olsen et al., 2016). The process of eelgrass' sexual reproduction, which can maintain the population's genetic diversity, has been well-described (Decock, 1980; Meling-Lopez and Ibarra-Obando, 1999; Larkum et al., 2005; Kendrick et al., 2012). The sexual cycle, through pollination and flowering, produces eelgrass seeds with genes that are different from their parental generation (Decock, 1980; Ort et al., 2012). Seed dispersal, following the processes of seed production and release, plays an important role in colonizing new habitats or recolonizing completely destroyed areas by virtue of the long seed dispersal distance of up to 150 km (Howe and Smallwood, 1982; Harwell and Orth, 2002; Kallstrom et al., 2008). *Z. marina* seeds become buried within the sediment, thus forming a seed bank (Fishman and Orth, 1996; Delefosse and Kristensen, 2012). Successful seedling establishment and survival, following some period of dormancy and seed germination, will enhance the eelgrass population's genetic diversity, which can increase their resilience to disturbance. The asexual reproduction of seedlings and overwintering shoots will rapidly increase shoot density through clonal growth. The eelgrass meadows will successively enter the next flowering period, and the life cycle continues.

The sexual reproduction of eelgrass from different populations has been discussed in many studies, and considerable variations in the temporal and spatial patterns of the sexual reproductive process were found in different populations and geographical regions. Eelgrass reproduction is greatly influenced by local environmental factors, which also impact their dispersal and recovery rate in established habitats (Decock, 1980; Churchill, 1983; McMillan, 1983; Phillips et al., 1983; Silberhorn et al., 1983; Hootsmans et al., 1987; van Lent and Verschuure, 1994; Meling-Lopez and Ibarra-Obando, 1999; Olesen, 1999; Hemminga and Duarte, 2000; Walker et al., 2001; Larkum et al., 2005; Ackerman, 2006; Morita et al., 2007). However, most of the studies were conducted in laboratories (Hootsmans et al., 1987; Pan et al., 2011; Xu et al., 2016), and some crucial processes in sexual reproduction are poorly understood (e.g., seedling establishment; Orth et al., 2012). Furthermore, there is limited datum on the contribution of sexual reproduction to the population recruitment of natural *Z. marina* L. (Cabaco and Santos, 2009, 2012; Furman et al., 2015).

Understanding population recruitment is essential for the conservation, management and restoration of *Z. marina*. Because of the lack of information on recruitment in *Z. marina* populations worldwide, populations in Swan Lake (SLL) and Huiquan Bay (HQB) from the north of China were chosen in the present study. We hypothesized that contribution of sexual reproduction to population recruitment would depend on geographic location; and seeds from different geographic locations would have different laboratory germination characteristics pertaining to local adaptability; and local environmental conditions, especially temperature regime, would induce shifts in sexual recruitment strategies. In the present study, we conducted a field survey of *Z. marina* reproduction in SLL and HQB. Additionally, the seed bank sizes at the two sites were investigated. We also investigated natural seed germination and seedling establishment. The aim of this study was to understand (1) the temporal dynamics of asexual and sexual reproduction in *Z. marina* at the two sites, and (2) the contribution of sexual reproduction in the population recruitment of these two populations. With these aims, the sexual reproduction of *Z. marina* was investigated using permanent quadrats and random sampling methods. In addition, the seed germination of the two populations was examined in the laboratory to validate and explain the field results. Our work will provide fundamental information for understanding the role of sexual reproduction in population recruitment and for forming restoration and conservation strategies.

## Materials and methods

### Study sites

**Figure 1:**
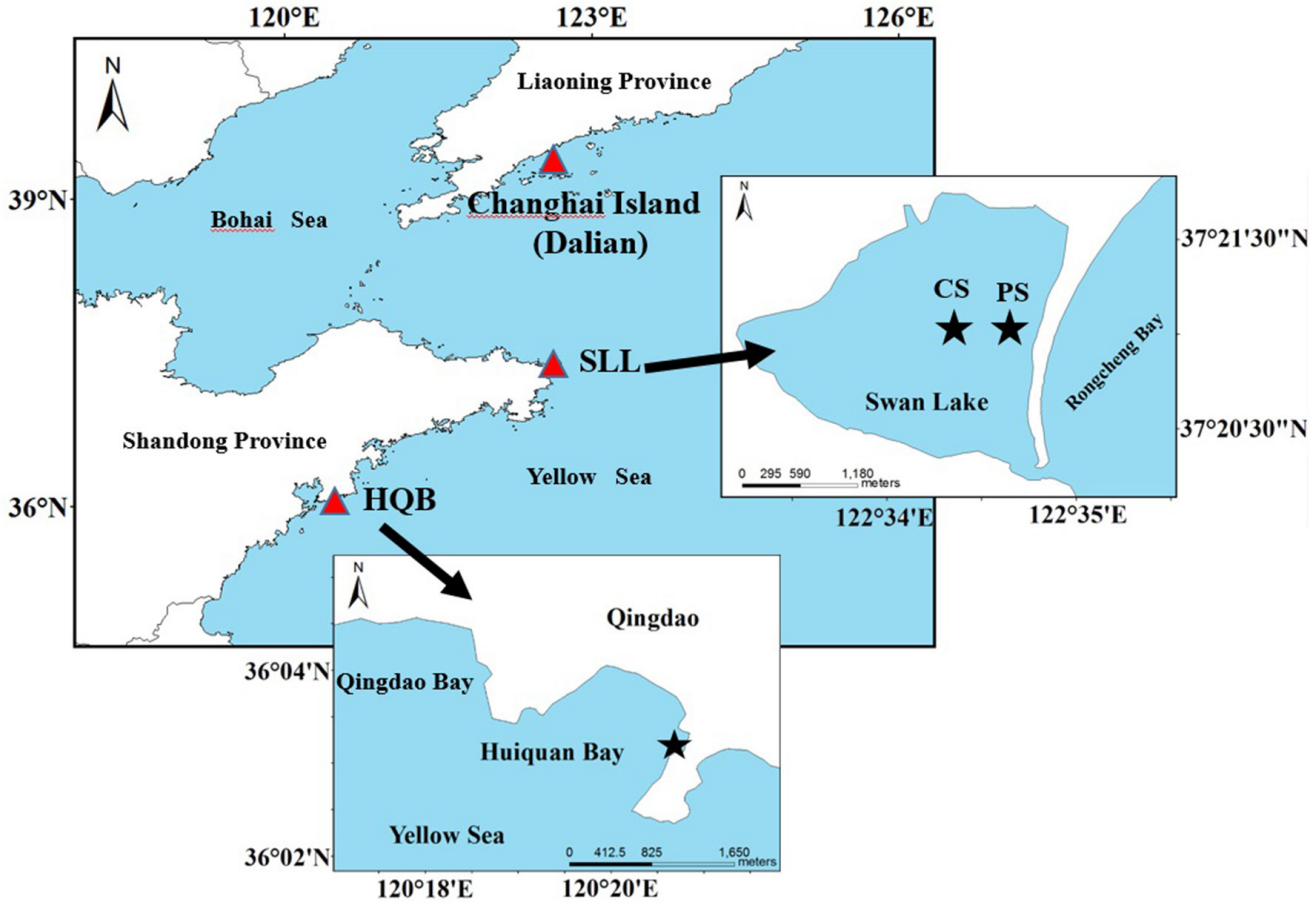
*Zostera marina* study sites of Swan Lake (SLL) and Huiquan Bay (HQB) as well as location of Changshan Island (Dalian). Red triangular symbols represent the locations of seagrass beds. CS (Center site) and PS (Patch site) represent the center of continuous distributed area and patch area of eelgrass in SLL, respectively.

Swan Lake (SLL; 37°21′N, 122°34′E) is marine lagoon northeast of Rongcheng City, northern China (Figure 1). It is approximately 2.1 km long and 1.8 km wide, with an area of ~4.8 km^2^. The lagoon is connected to the Rongcheng Bay by a narrow mouth, which is 86 m in width. It is protected from currents and waves by a long and narrow eastern sandbar. The lagoon experienced irregular semidiurnal mixed tides, with a mean tidal range of ~0.9 m. The annual concentrations (mean ± *sd*) of ammonia, nitrate, nitrite and phosphate in SLL were 2.14 ± 1.29, 1.31 ± 1.53, 0.15 ± 0.10, and 0.28 ± 0.17 μmol L^−1^, respectively (Zhou et al., 2015). The lagoon is much shallow, with an average depth <1.5 m. The sediment of SLL is mainly sandy. It functions as a suitable habitat for seagrasses (Zhang et al., 2014, 2015; Zhou et al., 2015; Xu et al., 2017). The lagoon, a national nature reserve of whooper swan in China, provides food resources and the largest wintering habitat for the whooper swan *Cygnus cygnus* in Asia (Wang et al., 2017). Eelgrass coverage is ~1.5 km^2^ (Zhou et al., 2015). We surveyed two study sites in the lagoon based on the eelgrass' spatial distribution (Figure 1). The center site was selected to represent the continuous area of eelgrass, and the patch site was located in a non-continuous patch area of the species. The aim of the selection of the center and patch sites was to investigate whether seagrass distribution (continuous area and non-continuous patch) has an influence on plant reproductive effort.

Huiquan Bay (HQB; 36°03′N, 120°20′E) is an open bay in Qingdao City, northern China (Figure 1). The tides are regular semidiurnal, with a mean tidal range of ~4.8 m. Annual concentrations (mean ± *sd*) of ammonia, nitrate, nitrite and phosphate were 3.81 ± 2.16, 5.87 ± 3.06, 0.68 ± 0.59, and 0.18 ± 0.09 μmol L^−1^, respectively (Liu, 2012). With the direct influence of the oceans on the climate, this area is the equivalent of a marine climate zone. The sediment of the bay is mainly sandy. The south-eastern corner of this bay, which is dominated by a seagrass meadow, is protected from currents and waves by the sandbar south of the bay. The seagrass meadow is a mix of *Z. japonica* and *Z. marina*.

### Environmental variables

Water temperature (°C) of SLL and HQB was measured every 15 min from July 2014 to December 2015 using HOBO data loggers (USA). Salinity was measured monthly at water surface using YSI Pro 30 (USA). During the study period, light intensity at canopy level was captured by an ECO-PAR sensor deployed in the seagrass bed. Daily photosynthetic photon flux densities (PPFD; mol photons m^−2^ d^−1^) were calculated as the sum of the quantum flux within a 24-h period. Three sediment cores (Diameter = 10.3 cm and depth = 10 cm) were collected at each location for determination of grain size distribution, based on laser diffraction analysis (Short and Coles, 2001).

### General clonal growth of population adult shoots

From July 2014 to January 2016, a random sampling method using a 20 × 20 cm quadrat was employed to investigate the adult shoot density (shoots m^−2^), shoot height (cm), and above and belowground biomass (g m^−2^) in SLL, which was designed to assess the general clonal growth dynamics of the adult shoots. A total of five samples were collected at each site in SLL. For HQB, five sampling events were conducted in March, June, August, November (2015) and January (2016), and a total of five samples were collected in each sampling event. The samples from SLL and HQB were filtered through a sieve (2 mm) carefully *in situ*, and the shoots were taken back to the laboratory for further treatments. The number of shoots was determined, and shoot height and above and belowground biomass in fresh weight were measured.

### Plant reproductive effort

Flowering shoot density and height of *Z. marina* were recorded using a 20 × 20 cm quadrat in SLL. The flowering shoots were harvested in SLL and HQB during July 2015 at the local species' flowering peaks and transported to the laboratory. The plant's reproductive effort was estimated by counting flowering shoot density, the number of spathes per flowering shoot and the number of seeds per spathe. The potential seed output per reproductive shoot and per unit area were calculated based on the plant's reproductive effort. The lengths and diameters of 184 seeds from each site were measured using an industrial digital camera (precision of 0.01 mm), and seed volumes were calculated based on the volume of an ellipsoid, π*/6* L D^2^ (Delefosse et al., 2016). Sixty seeds were randomly selected and divided into three replicates. Seeds were spread to dry on soft paper towels to remove water, and the wet weight of seeds was measured.

### Seed banks

From September 2014 to November 2015, 6–7 samples were randomly collected monthly at two sites with a PVC tube (19-cm diameter × 12-cm depth) in SLL. Samples were passed over a 5-mm sieve to remove large detritus, and detritus material was separated from eelgrass seeds through a 0.7-mm sieve, and then the remaining material in the sieve was taken back to the laboratory for analysis. The seeds were selected from the material, and then pressed using tweezers. The rotten seeds and seed coats would be flat. The intact seeds were selected out for counting seed bank density. The viable seeds were referred to the intact seeds in this paper.

For HQB, three transecting areas, running vertical to the shore and at a length of 35–70 m from the upper limit to the lower limit of the whole meadow, were established to investigate seed bank sizes. Samples were collected at 5–7 m intervals along the transecting lines. From October 2015 to September 2016, four sampling events in HQB were conducted for comparison with surveys in SLL. In addition, seven samples were randomly collected in October 2014 and November 2016 in HQB.

### Seed germination and seedling development

Seed germination in the field was defined as the emergence of the cotyledon from the sediment surface. This was easier to determine and is a more ecologically meaningful metric than quantifying the splitting of the seed coat alone (Churchill, 1983; Brenchley and Probert, 1998). The random sampling and permanent quadrat survey methods were employed to investigate the processes of seed germination and seedling survival in SLL and HQB.

Random sampling method: from March to June 2015, 5 cores were sampled twice a month using a PVC tube (19-cm diameter × 12-cm depth) at both patch and center sites in SLL. The samples were sieved (2-mm) carefully *in situ*, and the retained seedlings and overwintering shoots were transported to the laboratory to count the number of seedling shoots and shoots per seedling and to measure shoot height. For HQB, the random sampling method was employed to assess densities of the seedlings by 5–10 quadrats (1-m × 1-m) *in situ* in January, June, December (2015) and January (2016). Seedlings and overwintering shoots were verified through the rhizomes and/or the original seed coats. After July, because seedlings were hard to distinguish from overwintering shoots, there was no datum on the seedlings.

Fixed sample method: 6 randomly selected 20 × 20 cm permanent quadrats were established in HQB on 23 March 2015 to monitor changes in eelgrass seedling height until June, and the seedling height were measured *in situ*.

### Sexual recruitment contribution of *Z. marina* population

In the beginning, seedlings could be easily distinguished from adult shoots through rhizomes and/or original seed coats. However, seedlings would be more and more difficult to be distinguished with the growth of seedling. In this study, sexual recruitment contribution (*SRC*; %) of *Z. marina* population was determined in the last survey when seedlings could be distinguished from adult shoots. The *SRC* was calculated using the following equations:

$$\begin{array}{l} SRC\left(\%\right)=\frac{D_{S}}{D_{S}+D_{A}}\times 100\%=\frac{D_{S}}{D_{T}}\times 100\%;\text{where}\\ D_{T}=D_{S}+D_{A} \end{array}$$

*D*_*S*_ = Density of seedling shoots (shoots m^−2^), *D*_*A*_ = Density of adult shoots (shoots m^−2^), and *D*_*T*_ = Density of total shoots (shoots m^−2^).

### Laboratory seed germination experiment

To investigate the effects of the seed source on germination, tests were conducted in the laboratory during October and November 2016. Seed germination in the experiment was explicitly defined as, not just the rupture of the seed coat, but also the emergence and growth of the cotyledon (Churchill, 1983; Brenchley and Probert, 1998). The seeds from reproductive shoots were harvested at each site during July 2016 and stored separately in high salinity artificial seawater (salinity 50; Pan et al., 2011) at 5°C (Zarranz et al., 2010; Dooley et al., 2013) until the germination experiments were conducted.

Seeds from SLL and HQB were both cultivated in the laboratory at a salinity level of salinity 30 at optimal germination temperatures of 10 and 15°C. Seeds were placed in 12-cm plastic Petri dishes containing 50 ml of artificial seawater (salinity 30) and 50 seeds each were exposed to temperatures of 10 and 15°C for a period of ca. 5 weeks, with three replicate Petri dishes per treatment. The numbers of germinated seeds were counted every 1–7 days. Petri dishes were placed in an incubator, and the artificial seawater was changed every 3 days. Temperatures were selected based on a previous study (Xu et al., 2016). The germination percent (GP) and germination value (GV(D)) were calculated to evaluate the differences in seed germination among the populations of SLL and HQB. The GP and GV(D) of the *Z. marina* seeds were calculated using the following equations:

$$\begin{array}{l} GP\left(\%\right)=\sum \frac{n_{i}}{N_{0}}\;\times 100\%;\\ GV\left(D\right)=\frac{\left(\sum DGS\right)}{N}\times \;GP\times \;10;\text{where}\\ DGS\;=\frac{G_{i}}{i} \end{array}$$

*n*_*i*_ = the number of seeds germinated in the *i*th day, *N*_0_ = the total number of seeds, *N* = total germination days, and *G*_*i*_ = the number of germinated seeds during the *i*th day.

### Data and analysis

Values are represented as means ± *sd*. Seasonal differences in biological and environmental variables were tested using a one-way analysis of variance (ANOVA) or independent *t*-test. A one-way ANOVA or *t*-test was also employed to compare the two sites (SLL and HQB). In the laboratory seed germination analysis, a two-way ANOVA was used to compare the effects of temperature and seed source on seed germination. When the interaction was significant, a one-way ANOVA or independent *t*-test was used to compare the temperature effect at each site and the seed source effect at each temperature (Zar, 1998). The homogeneity of variance was tested using Levene's test (Zar, 1998). SPSS 20.0 for Windows 8.1 was used for all data analyses. Differences were considered significant at a probability level of *p* < 0.05.

## Results

### Environmental variables

**Figure 2:**
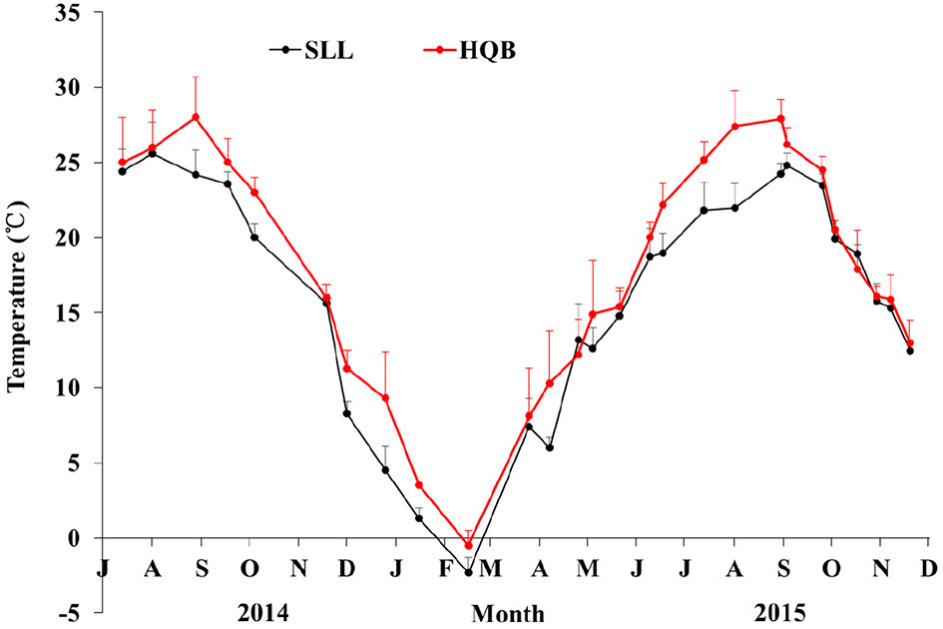
Water temperatures of Swan Lake (SLL) and Huiquan Bay (HQB). Red line represents the temperature of HQB; black line represents water temperature of SLL. Values are means ± *sd*.

**Figure 3:**
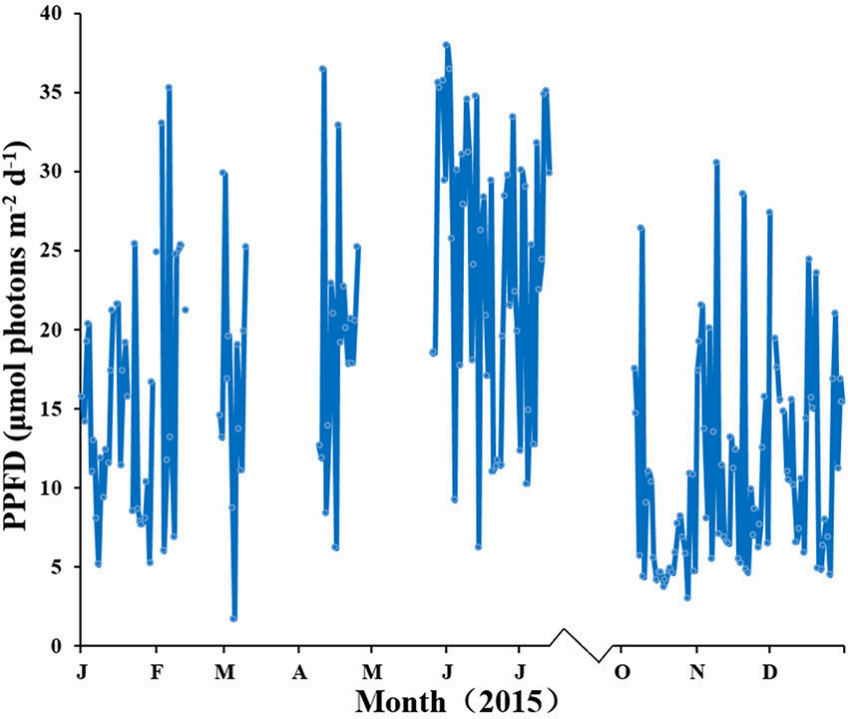
Light intensity of the canopy of *Zostera marina* in Swan Lake (SLL) during the study period.

**Figure 4:**
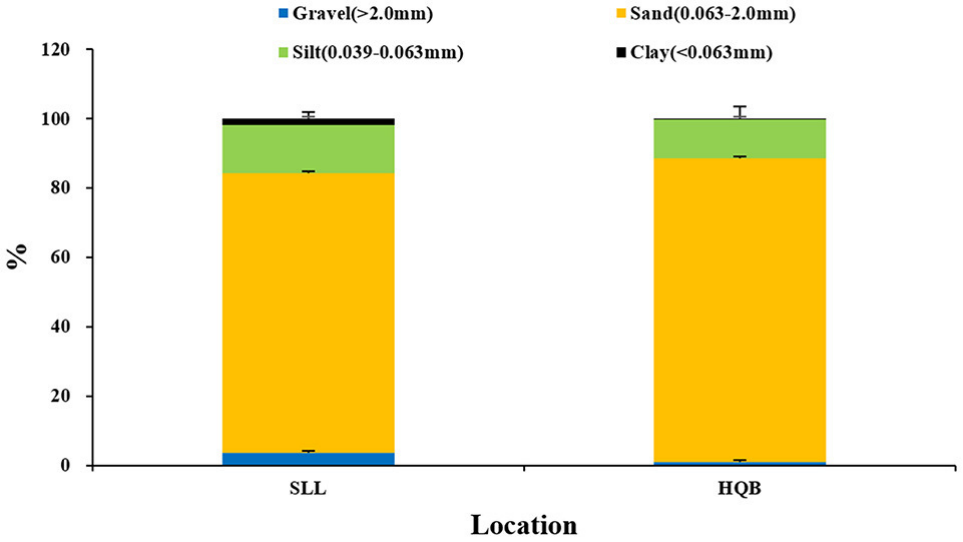
Sediment grain sizes at Swan Lake (SLL) and Huiquan Bay (HQB).

Water temperature showed a clear annual pattern at both SLL and HQB (Figure 2). The temperature ranged from −2.30°C (February) to 25.60°C (August) at SLL, and the annual average water temperature of the lagoon was 16.13 ± 7.76°C. The temperature ranged from −0.50°C (February) to 28.00°C (August) in HQB, and the annual average water temperature is 17.94 ± 7.80°C. HQB had higher water temperatures than SLL because HQB had much warmer winters and returned to spring temperatures more quickly. The salinity ranged from 31.3 to 33.7 in SLL, and 31.6 to 32.7 in HQB. The photosynthetic photon flux densities (PPFD) showed a clear annual pattern at SLL (Figure 3). The PPFD increased from January and reached highest in summer months (June-July). The PPFD at HQB was similar to that in SLL. The sediments at both SLL and HQB (Figure 4) were mainly composed of sands (0.063–2.0 mm), but the proportion of sands at SLL were significantly lower than that at HQB (80.68 ± 1.45% vs. 87.64 ± 4.70%).

### Vegetative shoot production

**Figure 5:**
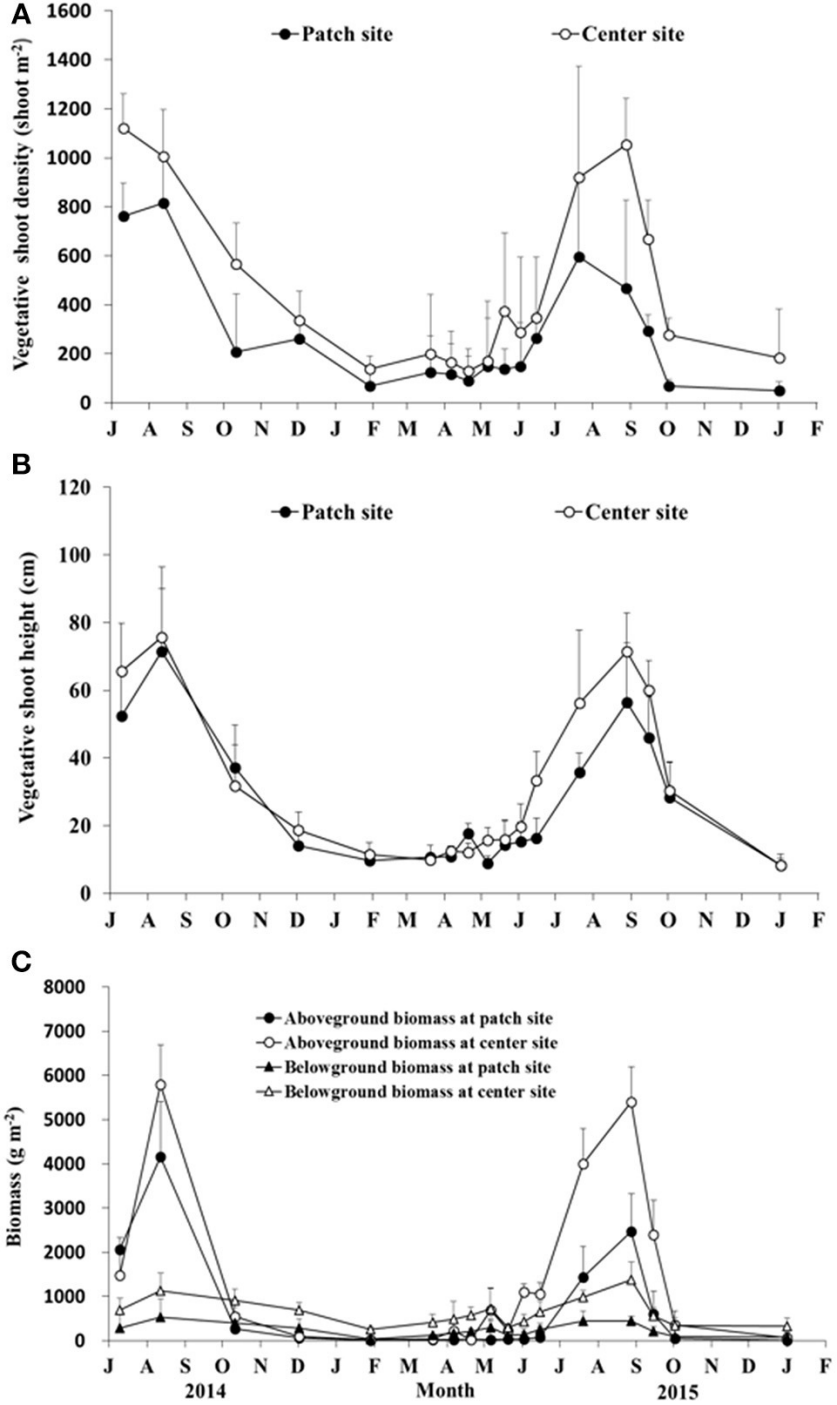
Temporal changes in the vegetative shoot density **(A)**, shoot height **(B)**, and biomass **(C)** of *Zostera marina* in Swan Lake. Values are means ± *sd*.

Densities of adult vegetative shoots at SLL was significantly affected by month (Patch site: *F* = 9.888, *df* = 77, *P* < 0.001; Center site: *F* = 13.522, *df* = 81, *P* < 0.001), and the mean adult density of the patch site was less than that of the center site (Figure 5A). The density at the center site declined gradually from 1,120.00 ± 140.19 shoots m^−2^ (July 2014; the maximum density) to 129.32 ± 90.35 shoots m^−2^ (April 2015), and then increased to 1,054.43 ± 187.42 shoots m^−2^ (August 2015). The density at the patch site had a similar trend. The maximum density was found in August 2014 (815.56 ± 182.51 shoots m^−2^), and the density in January 2016 was the lowest, at 49.38 ± 35.17 shoots m^−2^. The shoot height at SLL also had a significant monthly pattern (Patch site: *F* = 73.481, *df* = 293, *P* < 0.001; Center site: *F* = 90.798, *df* = 338, *P* < 0.001), and the changes in height had a similar trend as the adult density (Figure 5B). Figure 5B also shows that the height of adult shoots were lower in the winter and spring and higher in the summer and autumn. The aboveground biomass was greater in the summer and lower in the winter (Figure 5C). The highest aboveground biomass at patch and center site were 4,164.62 ± 1,238.26 and 5,794.27 ± 890.14 g m^−2^ (August 2014), respectively.

For HQB, the adult shoot density also showed significant seasonal patterns (*F* = 45.752, *df* = 14, *P* < 0.001), with a peak in the summer (1,118.89 ± 152.05 shoots m^−2^) and a trough (233.33 ± 19.25/262.96 ± 46.26 shoots m^−2^, 2014/2015, respectively) in the winter (Table 1). The shoot height at HQB also had a seasonal pattern (*F* = 6.311, *df* = 244, *P* < 0.001), The above and belowground biomass showed significant seasonal patterns (*F* = 27.015/25.739, *df* = 15/15, *P* < 0.001). The highest above and belowground biomass were 3,689.39 ± 1,008.84 and 2,832.55 ± 568.96 g m^−2^ (*P* < 0.05; Table 1).

**Table 1:** The shoot density, shoot height, and aboveground and underground biomasses of *Zostera marina* in Huiquan Bay.

**Date**	**Adult shoot density (shoots m^−2^)**	**Shoot height (cm)**	**Aboveground biomass (g m^−2^)**	**Underground biomass (g m^−2^)**
7-Mar-15	233.33 ± 19.25^a^	35.93 ± 12.66^b^	319.26 ± 110.29^c^	395.93 ± 164.54^b^
6-Jun-15	487.50 ± 88.39^c^	64.31 ± 13.09^a^	*1, 015.67*±103.00^b^	820.83 ± 543.67^b^
31-Aug-15	*1, 118.89*±152.05^d^	80.23 ± 13.83^a^	*3, 689.39*±*1, 008.84*^a^	*2, 832.55*±568.96^a^
30-Nov-15	444.44 ± 101.84^bc^	29.98 ± 10.73^b^	437.04 ± 336.66^c^	701.86 ± 101.65^b^
1-Jan-16	262.96 ± 46.26^ab^	35.85 ± 11.49^b^	332.59 ± 87.78^c^	422.59 ± 108.81^b^

*Values are means ± sd. Different letters denote significant differences (p < 0.05)*.

### Plant reproductive effort

**Figure 6:**
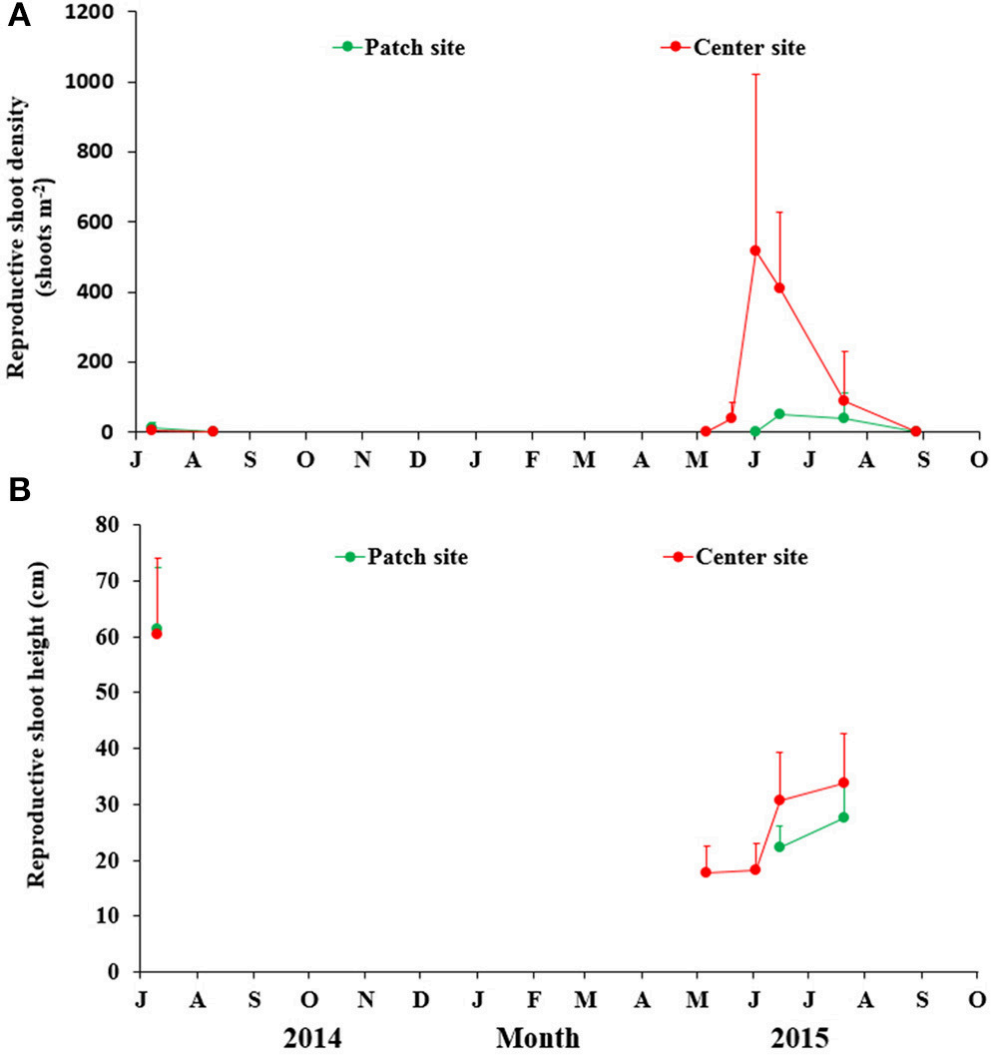
Temporal changes in the reproductive shoot density **(A)** and height **(B)** of *Zostera marina* at the two study sites in Swan Lake. Values are means ± *sd*.

The flowering shoots in SLL were first observed in early May at the center site, and the density rose gradually to peak at the beginning of June (517.27 ± 504.29 shoots m^−2^) with a flowering percent of 48.99 ± 29.79%. However, eelgrass flowering shoots only lasted from June to July at the patch site. At both sites, the flowering shoot density decreased quickly and disappeared completely by the end of July. Densities of flowering shoots at the center site were higher than those at the patch site (Figure 6A).

The reproductive shoot height at the patch site was significantly affected by month (*F* = 26.590, *df* = 12, *P* < 0.001), and the flowering shoot height in July 2014 was significantly higher than in June and July of 2015 (*P* = 0.003 and 0.002, respectively). The shoot height during flowering at the center site also changed over the months (*F* = 40.289, *df* = 69, *P* < 0.001), and the flowering shoot height in July (2014) was significantly higher than May, June (2nd and 15th) and July in 2015 (*P* = 0.001, 0.001, 0.005, and 0.011, respectively). There was no significant diference between the patch and center sites (*t* = −1.830, *df* = 42, *P* = 0.075; Figure 6B).

Densities of HQB reproductive shoots peaked in the start of June with 203.04 ± 116.50 shoots m^−2^ and a 29.40% flowering percent (Table 2). HQB flowering shoot density was not statistically different from that of SLL (*t* = 1.358, *df* = 9, *P* = 0.240; Table 2). HQB flowering shoots were first observed in the middle of April. Flowering shoot density did not increase rapidly until the start of June, but quickly ebbed after July. Flowering had stopped by the end of July.

**Table 2:** Reproductive effort and seed sizes of *Zostera marina* from Swan Lake and Huiquan Bay.

	**Swan Lake**	**Huiquan Bay**	***P***
**REPRODUCTIVE EFFORT**			
Density of flowering shoots (shoots m^−2^)	517.27 ± 504.29	203.04 ± 116.50	0.240
No. spathes per flowering shoot	10.83 ± 3.31^a^	21.5 ± 5.32^b^	0.002
No. seeds per spathe	9.57 ± 3.44^a^	7.98 ± 3.73^b^	0.020
No. seeds per flowering shoot	103.67 ± 37.95	142.83 ± 52.49	0.169
No. seeds per square meter	53,623.66 ± 19,628.11^a^	29,000.88 ± 10,657.89^b^	0.022
**SEED PARAMETERS**			
Seed weight (mg ind^−1^)	5.78 ± 0.34^a^	8.81 ± 0.03^b^	0.004
Seed length (mm)	3.13 ± 0.21^a^	3.70 ± 0.22^b^	<0.001
Seed diameter (mm)	1.60 ± 0.13^a^	1.81 ± 0.12^b^	<0.001
Seed volume (mm^3^)	44.71 ± 8.92^a^	67.13 ± 10.52^b^	<0.001

*Values are means ± sd. Different letters denote significant differences (p < 0.05)*.

The reproductive characteristics of the flowering shoots and the seed parameters of *Z. marina* at SLL differed to those at HQB (Table 2). The number of spathes per flowering shoot was significantly greater in the population in HQB (21.5 ± 5.32 spathes flowering shoot^−1^) than in SLL (10.83 ± 3.31 spathes flowering shoot^−1^; *t* = 4.170, *df* = 11, *P* = 0.002). The number of seeds per spathe was significantly greater in the population of SLL (9.57 ± 3.44 seeds spathe^−1^) than of HQB (7.98 ± 3.73 seeds spathe^−1^; *t* = −2.361, *df* = 171, *P* = 0.020). The potential seed production of SLL and HB were estimated to be 53,623.66 ± 19,628.11 seeds m^−2^ and 29,000.88 ± 10,657.89 seeds m^−2^, with the former being significantly greater than the latter (*t* = 2.700, *df* = 11, *P* = 0.022).

The values of seed weight and size (length, diameter and volume) of HQB were significantly greater than those of SLL (Table 2). The seeds from HQB (8.81 ± 0.03 mg) were heavier than the seeds from SLL (5.78 ± 0.34 mg; *t* = −15.158, *df* = 5, *P* = 0.004), and the seed length and diameter of HQB were also greater than those of SLL (*t* = 6.172/17.304, *df* = 369, *p* < 0.001; Table 2). Thus, the seed volume of HQB was significantly greater than that of SLL (*t* = 15.463, *df* = 369, *p* < 0.001).

### Seed banks

**Figure 7:**
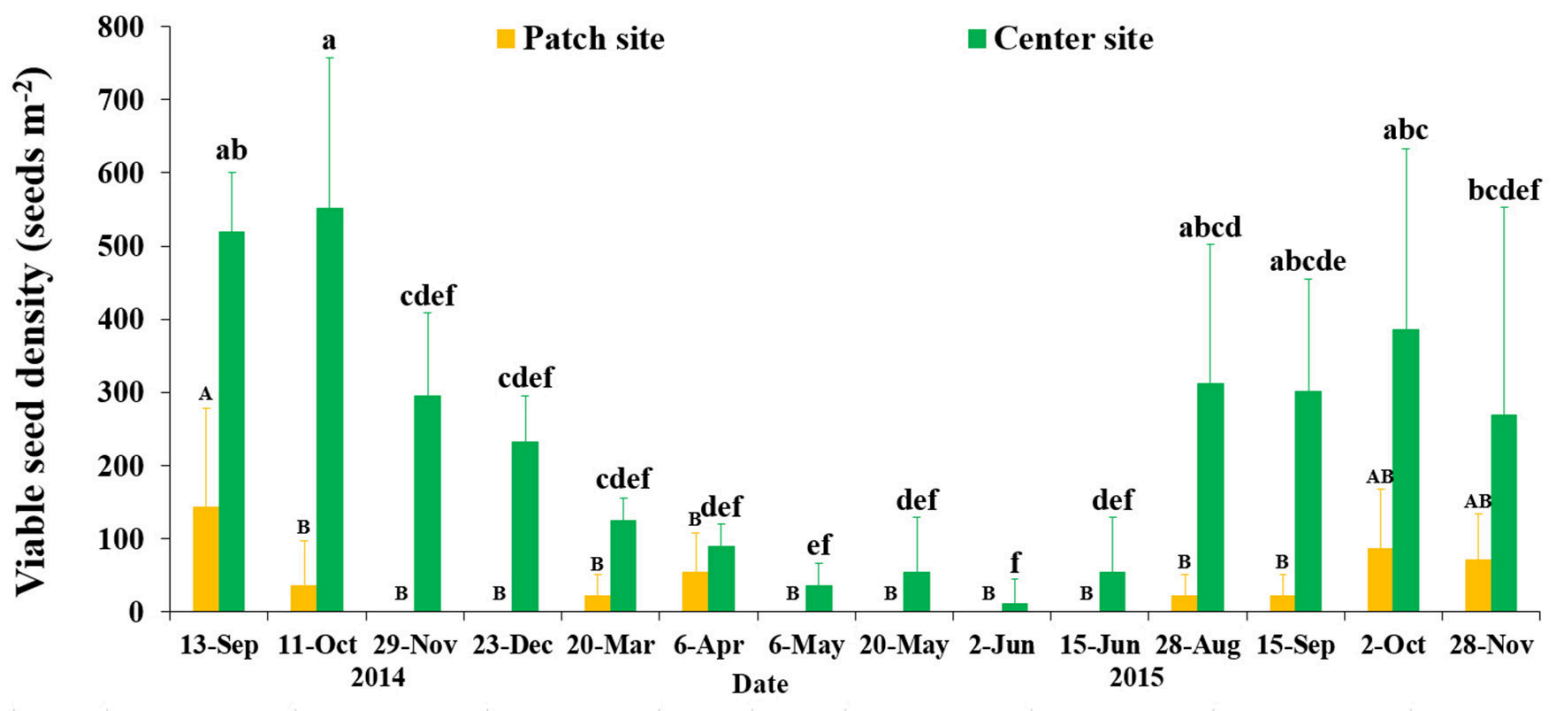
Temporal changes in the seed bank densities of *Zostera marina* at the two study sites in Swan Lake. Values are means ± *sd*. Different capital or small letters denote significant differences (*p* < 0.05).

Eelgrass seeds were dispersed heterogeneously in SLL, and in our investigation seed densities showed a clear temporal pattern with substantial variances at both the patch and center sites (*F* = 2.762/3.014, *df* = 54/59, *P* = 0.007/0.003, respectively; Figure 7). Mean seed densities at the center site was greater than that at the patch site. Seed densities at the center site increased to 552.21 ± 204.94 seeds m^−2^ (Oct 2014) when the flowering shoots disappeared and the seeds were buried in sediment of SLL. We observed that seeds were shed in July, and seed densities increased over the following several months until Oct 2015, when seed densities at the center site peaked at 386.55 ± 247.19 seeds m^−2^, and the seed burial rate was only 0.72% according to the predicted value of seed production at the center site. Seed densities started to decline after seed burial occurred in November. The decline in viable seeds was very high, at 57.87% (determined by the mean values in October and December 2014) until mass seed germination occurred in middle March of the next year. Seed densities declined dramatically and approached 0 from April to July (Figure 7). Comparing the examined plots over 2 years, no significant inter-annual variation was detected among the months of September, October and November (*P* > 0.05).

HQB seed bank densities declined from 4 October 2015 (254.35 ± 613.34 seeds m^−2^) to 30 October 2015 (43.64 ± 85.68 seeds m^−2^) and from August 2016 (329.71 ± 914.25 seeds m^−2^) to November 2016 (34.92 ± 56.08 seeds m^−2^; Table 3). Density of seed coats ranged from 172.35 ± 354.47 seeds m^−2^ (August 2016) to 638.90 ± 324.86 seeds m^−2^ (November 2016; Table 3).

**Table 3:** Densities of viable seeds and seed coats of *Zostera marina* L. in Huiquan Bay.

**Date**	**Viable seed (seeds m^−2^)**	**Seed coat (seeds m^−2^)**
8-Oct-14	0	6.71 ± 24.09
4-Oct-15	254.35 ± 613.34	265.41 ± 277.36
30-Oct-15	43.64 ± 85.68	48.68 ± 67.34
31-Aug-16	329.71 ± 914.25	172.35 ± 354.47
20-Sep-16	160.91 ± 457.36	526.32 ± 780.40
30-Nov-16	34.92 ± 56.08	638.90 ± 324.86

*Values are means ± sd*.

### Seed germination and seedling development

**Figure 8:**
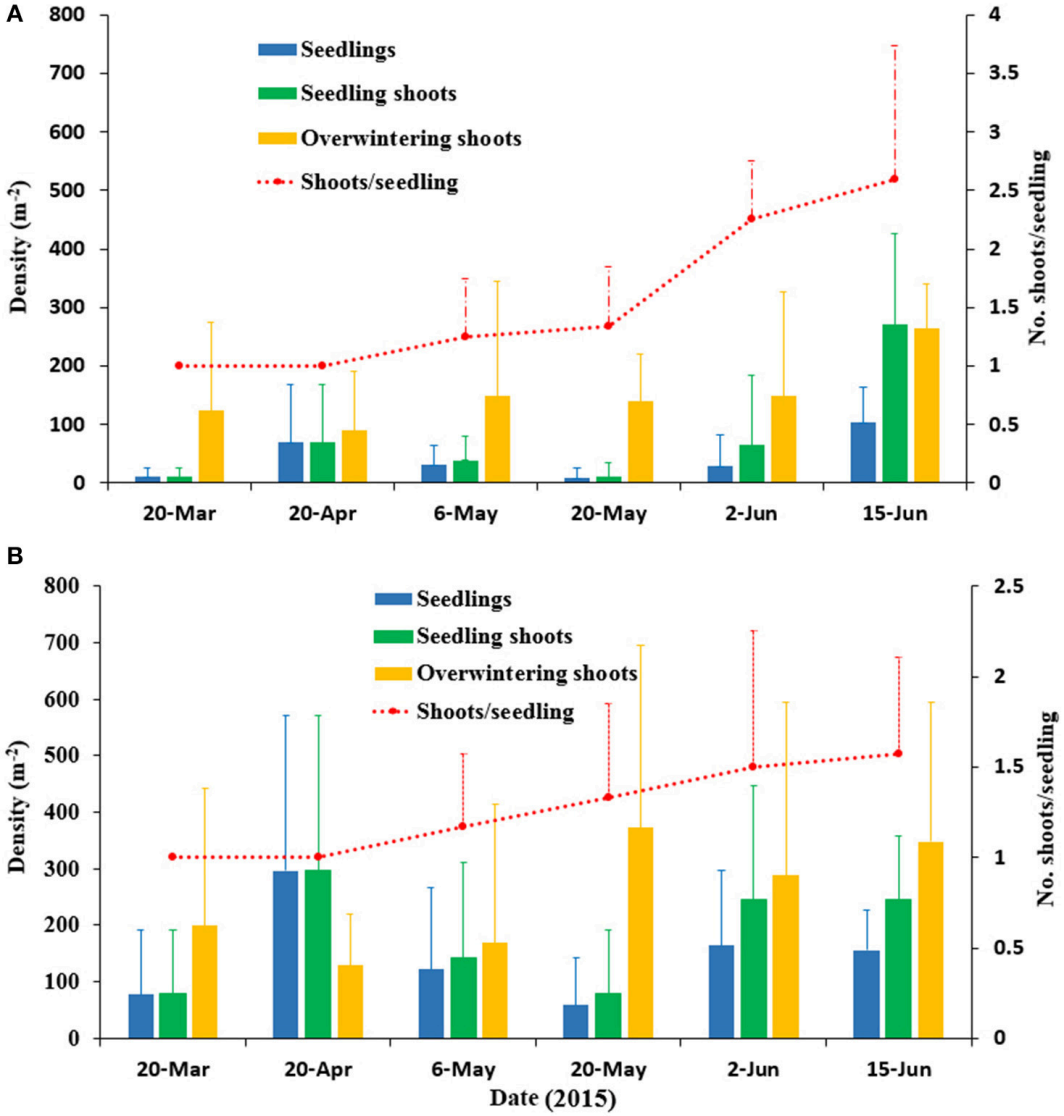
Densities of seedlings, seedling shoots and overwintering shoots, and the number of shoots per seedling of *Zostera marina* at the patch site **(A)** and the center site **(B)** in Swan Lake in 2015. Values are means ± *sd*.

Seed germination was documented in the spring in SLL, and there was no seed germination in the autumn. The seed germination in SLL lasted from March to the end of May. Seed germination ended when the water temperature rose to 14.8°C in May. When the temperature increased to 18.7°C (June), no new seed germination occurred. The seedling density increased dramatically and peaked in April with a maximum value 296.88 ± 274.27 seedlings m^−2^ at the center site (Figure 8). The seedlings began clonal growth in May and the number of shoots per seedling at the patch and center sites were 1.25 ± 0.5 and 1.16 ± 0.41, respectively, on 6 May and 2.60 ± 1.14 and 1.57 ± 0.53, respectively, on 15 June (Figure 8). Thus, the number of shoots per seedling at the patch site was slightly greater than the number at the center site. In the last seedling sampling, densities of seedlings and seedling shoots were 156.25 ± 71.32 seedlings m^−2^ and 245.54 ± 112.07 seedling shoots m^−2^, respectively, on 15 June 2015 at the center site. Densities of the adult shoots were 348.16 ± 247.03 shoots m^−2^. Thus, the *SRC* at the center site was 41.36%. Similarly, the *SRC* at the patch site was 50.52%.

**Figure 9:**
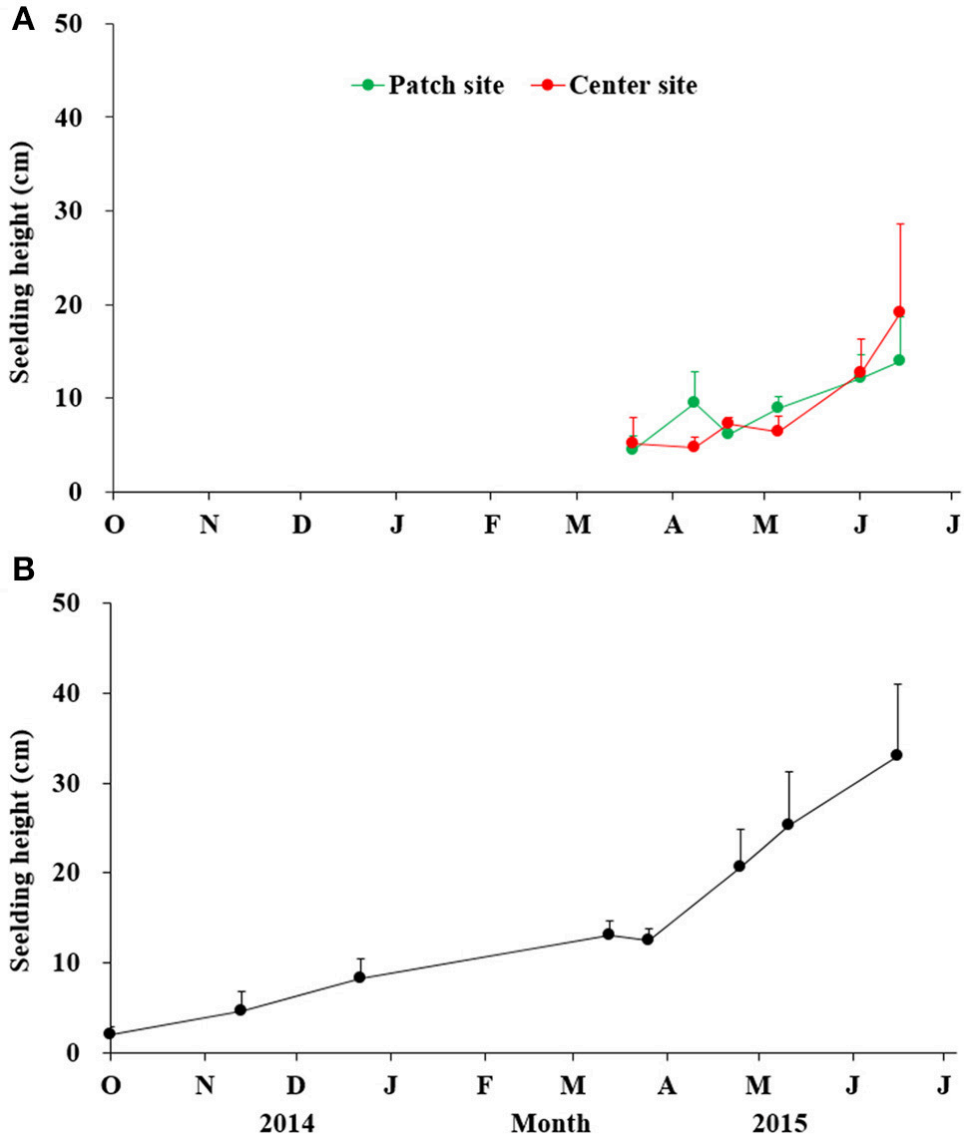
The seedling heights of *Zostera marina* in Swan Lake **(A)** and Huiquan Bay **(B)**. Values are means ± *sd*.

The variations of seedling height at two sites are shown in Figure 9A. The seedling height at both the patch and center sites varied significantly by date (patch site: *F* = 10.503, *df* = 36, *P* < 0.001; center site: *F* = 9.512, *df* = 43, *P* < 0.001), and there was no significant difference between the patch and center sites (*t* = −1.483, *df* = 80, *P* = 0.143; Figure 9A). With the temperature increase from 7.4°C (March 2015) to 19°C (June 2015), the seedling height at the patch site increased from 4.39 cm to 13.89 cm, and it increased at the center site from 5.08 cm to 19.14 cm. No new seedlings with shoot height of <10 cm germinated in the start of June.

At HQB, in the last servey on 6 June 2015 when seedling could be distinguished from adult shoots through rhizomes and/or original seed coats, densities of seedling shoots were 12.67 ± 7.02 seedling shoots m^−2^ (Table 4), and densities of adult shoots were 487.50 ± 88.39 shoots m^−2^. Thus, the *SRC* was 2.53%.

**Table 4:** Densities of seedlings, seedling shoots and the number of shoots per seedling of *Zostera marina* in Huiquan Bay during 2015–2016.

**Date**	**Density of seedlings (No. m^−2^)**	**Density of seedling shoots (No. m^−2^)**	**No. shoots per seedling**
4-Jan-15	6.00 ± 3.61^a^	6.00 ± 3.61^a^	1.00 ± 0.00^a^
7-Mar-15	–	–	1.00 ± 0.00^a^^*^
23-Mar-15	–	–	1.04 ± 0.11^a^^*^
21-May-15	–	–	1.57 ± 0.39^b^^*^
6-Jun-15	6.33 ± 2.08^a^	12.67 ± 7.02^a^	1.95 ± 0.78^b^
1-Dec-15	5.30 ± 5.70^a^	5.30 ± 5.70^a^	1.00 ± 0.00^a^
1-Jan-16	8.00 ± 2.35^a^	8.00 ± 2.35^a^	1.00 ± 0.00^a^

*Values are means ± sd. Different letters denote significant differences (p < 0.05)*.

* *Data were derived from fixed sample method*.

At HQB, which has a higher water temperature than SLL, seed germination occurred much earlier than at SLL. Seed germination started at the beginning of October when the seeds were buried in the sediment, and most seeds germinated during October and November, while we found only three seeds that germinated in the spring (March 2015) in the whole bed. HQB seedling density was <10 seedlings m^−2^, and there were no differences between January 2014 and January 2015 (Table 4). The clonal growth of seedlings began on 23 March, and increased quickly in May (water temperature >15°C; Figure 2) with 1.57 shoots per seedling by 21 May, then 1.95 shoots per seedling had grown by 6 June (Table 4).

The process of HQB seedling growth using fixed sample method is shown in Figure 9B. From 23 March to 26 June, the average height of the seedlings in the six permanent quadrats in HQB sharply increased from 12.49 ± 1.24 cm (April) to 33.05 ± 8.01 cm (June).

### Laboratory seed germination

**Figure 10:**
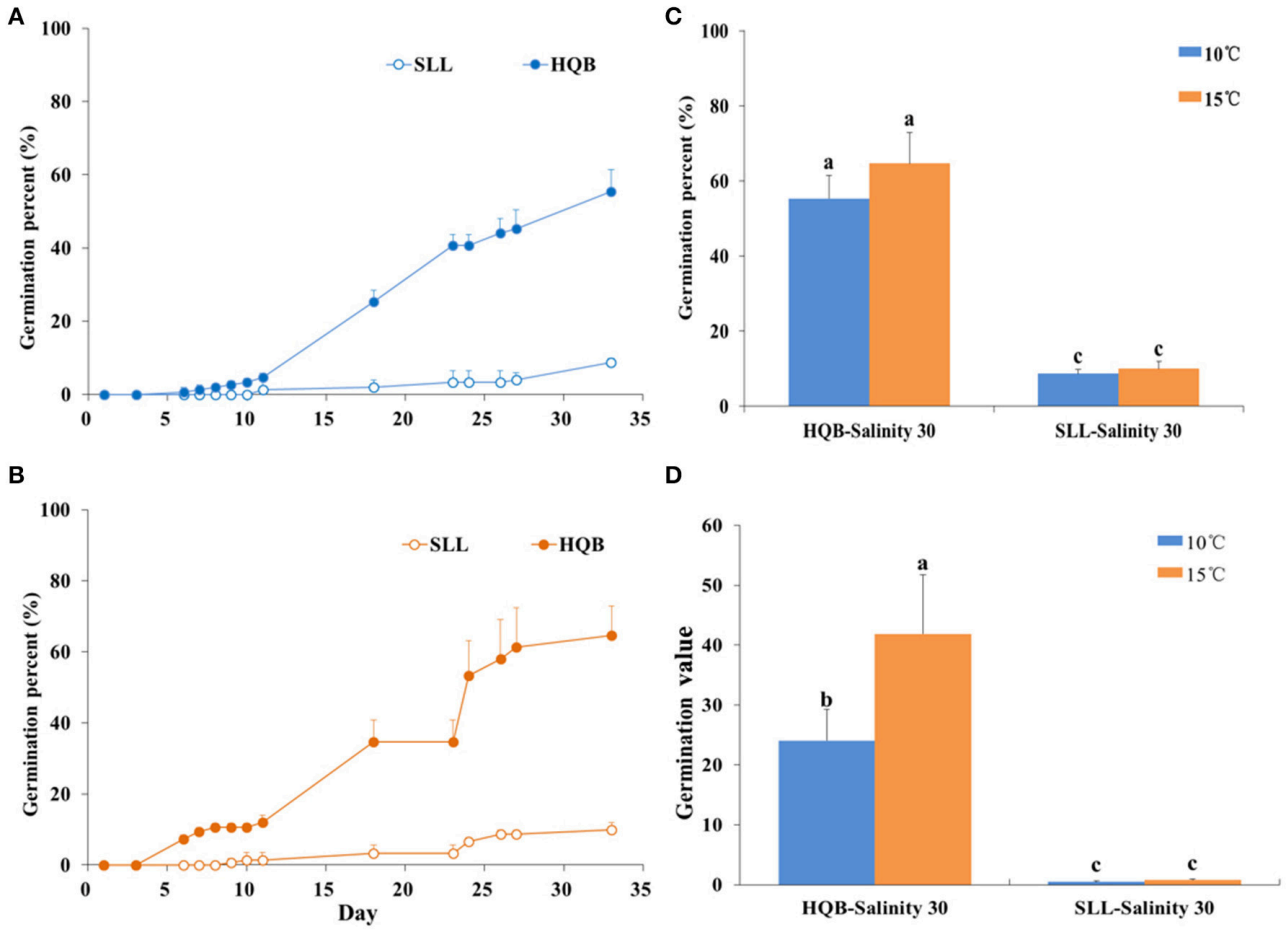
Comparison of the germination percent (GP) and germination value (GV(D)) of *Zostera marina* seeds from different sources, Swan Lake (SLL) and Huiquan Bay (HQB), subjected to two temperatures (10 and 15°C). **(A,B)** GP at 10 and 15°C, respectively; **(C,D)** GP and GV(D) after 4 weeks, respectively. Values are means ± *sd*. Different lowercase letters (a,b) in **(C)** and **(D)** denote significant differences (*p* < 0.05).

Germination percent (GP) curves of *Z. marina* seeds from two sources, SLL and HQB, subjected to the temperatures of 10 and 15°C are shown in Figure 10. The GP of the HQB seeds was always significantly greater than that of the SLL seeds (*P* < 0.001), regardless of temperature (Figure 10). The GP of the HQB seeds increased rapidly, while that of the SLL seeds increased much more slowly. Regardless of temperature, the GV(D) of HQB seeds was also greater than that of the SLL seeds (Figure 10).

## Discussion

**Figure 11:**
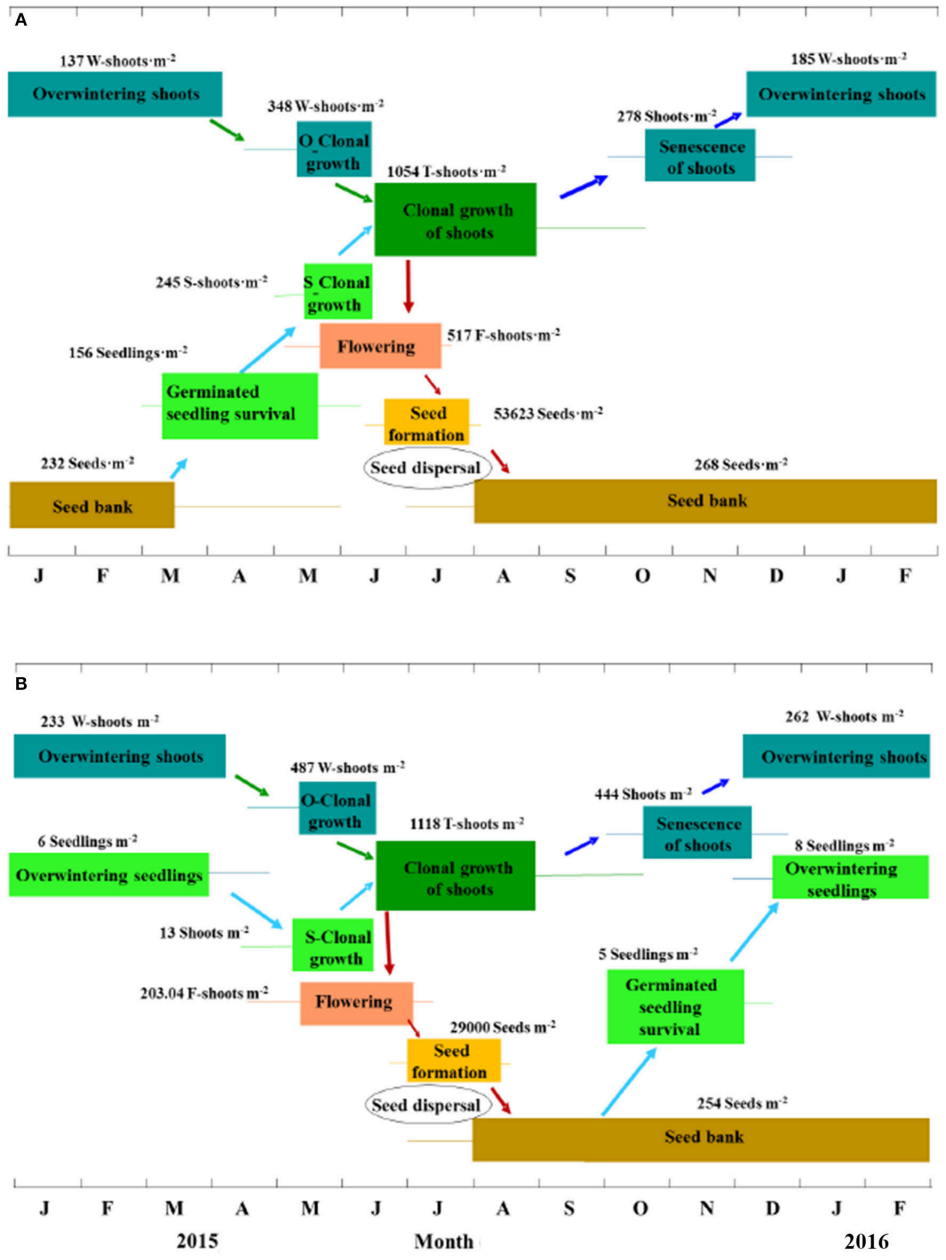
The reproduction and recruitment cycle of *Zostera marina* in Swan Lake **(A)** and Huiquan Bay **(B)**, China. The colored lines and rectangles represent the accidental and the main timing of different stages within the reproduction and recruitment cycle, respectively; O-Clonal growth and S-Clonal growth represent clonal growth of overwintering shoots and seedlings, respectively; W-shoots, S-shoots, T-shoots, and F-shoots refer to overwintering shoots and shoots from their clonal growth, seedling shoots, total shoots and flowering shoots, respectively. Values (means) are based on the investigation in 2015 and represent the peaks of each stage. Four processes are involved in this cycle: **(A)** i, seed germination from mid-March, seedling establishment beginning in April and completed at mid-June and seedling S-Clonal growth beginning in May; ii, W-shoot clonal growth beginning in mid-April; iii, rapid clonal growth of mixed S- and W-shoots beginning in June, flowering proceeding from May and seed dispersal mainly occurring in mid-July, and at the same time seeds are being dispersed into the sediment (seed bank); iv, shoots that did not flower decline with time and some of them overwinter, entering the next cycle. **(B)** i, seed germination from October, seedling establishment beginning from mid-October and finishing in mid-December, S-Clonal growth beginning in April; ii, W-shoots begin clonal growth at the same time; iii, rapid clonal growth of mixed S- and W-shoots beginning in June, flowering proceeding from mid-April and seed dispersal mainly occurring in mid-July, and at the same time seeds are being dispersed into the sediment (seed bank); iv, shoots that did not flower decline with time and some of them overwinter, entering the next cycle.

Eelgrass sexual reproduction and recruitment have received insufficient attention in the literature (Table 5). The present study was the first systematic survey on the life history from seed production to seedling recruitment of natural *Z. marina* populations. Based on the results of the present study, the reproduction recruitment cycle of a *Z. marina* population in SLL has been clarified into four processes and the key steps have been quantified. The four processes were as follows (Figure 11A): (1) seeds mainly germinated from middle of March until May and seedlings began clonal growth from May; (2) overwintering shoots began clonal growth from the middle of April, which was later than germination; (3) both seedling shoots and overwintering shoots began rapid clonal growth periods at the beginning of June, proceeded to flowering in early May and produced seeds from June to July, during which time mature seeds were dispersed into sediment to form a seed bank; (4) flowering shoots withered after reproduction, while vegetative shoots decayed with time and some of them overwintered, entering the next cycle.

**Table 5:** Comparison of sexual reproduction among *Zostera marina* populations from Swan Lake, Huiquan Bay, and other study sites.

**Region**	**Locations**	**Growth form**	**Flowering periods**	**Seed germination**	**Flowering shoot density (shoots m^−2^)**	**Seed production (seeds m^−2^)**	**Seed bank size (seeds m^−2^)**	**Seedling contribution percent (%)**	**References**
Northwestern Pacific	Swan Lake, China (FO)	Annual and perennial	May-Jul	Mar-May	517.27 ± 504.29 (Jun, 2014)	53,623.66 ± 19,628.11 (Jul, 2015)	552.21 ± 204.94 (Oct, 2014)	41.36	This study
	Huiquan Bay, China (FO)	Perennial	Apr-Jul	Sep-Nov	203.04 ± 116.50 (Jun, 2014)	29,000.88 ± 10,657.89 (Jul, 2015)	254.35 ± 613.34 (Sep, 2016)	2.53	
	Changhai County, China	Perennial	May-Jul	Mar-May	85.71 ± 122.51 (Jul, 2016)	5,112.73 ± 2,982.25 (Jul, 2016)	–	–	
	Jingdong Bay, Korea (FO)	Perennial	Jun (2002)	–	22.5 ± 3.9	5,544 (May, 2002)	–	22.6 (2002)	Lee et al., 2007
			Jan-Jul (2003)	Oct-	10.2 ± 6.1 (Apr, 2003)	1,265 (Apr, 2003)	200 (Aug, 2003)	100 (2003)	
			Feb-Aug (2004)	–	44.9 ± 9.7	15,872 (Apr, 2004)	850 (Sep, 2005)	6 (2004)	
			Feb-Aug (2005)	–	103.4 ± 14.4 (Apr, 2005)	25,476 (Apr, 2005)	1,780 (Jul, 2005)	8 (2005)	
	Odawa Bay, Japan	Perennial	Apr-Jun	–	50 (May, 1976)	–	–	–	Aioi, 1980
	Tategami, Ago Bay, Japan	Annual	Apr-Jun (2004)	–	–	6,000 ± 2,400 (May, 2004)	1,157 ± 360 (Oct, 2004)	–	Morita et al., 2007
	Hamajima, Ago Bay, Japan	Perennial	–	–	–	–	5 ± 9 (Oct, 2004)	–	
Northeastern pacific	Port Clarence, Bering Sea, USA	–	–	–	90 (Sep, 1967)	–	–	–	McRoy, 1970
	Safety Lagoon, Bering Sea, USA	–	–	–	100 (Sep, 1967)	–	–	–	
	Sawmill Bay, USA	–	–	–	3.5 (Jun, 1967)	–	–	–	
	Douglas Bay, USA	–	–	–	35.0 ± 79.0 (Aug, 1975)	392 (Aug, 1975)	–	–	Phillips et al., 1983
	Puget Sound, washington, USA	Perennial	Mar-Oct	Apr-Jul	66.0 ± 33.6 (Aug, 1964)	2,059 ± 253 (Aug, 1964)	–	–	Kentula, 1982; Phillips et al., 1983
	Netarts Bay, Oregon, USA	Annual and perennial	May-Sep	Feb-Jun	65 (Jun, 1980)	–	–	–	Kentula, 1982
	Concepcion Bay, Gulf of California, Mexico	Annual	-	-	555.0 ± 351.8 (Apr, 1974)	19,783 ± 619 (Apr, 1974)	–	–	Phillips et al., 1983
	Bahia Kino, Gulf of California, Mexico	Annual	Mar-Jul	Oct-early Nov	2,636 ± 242.7 (Mar, 1977)	11,132–100.740 (Mar, 1977)	–	–	Phillips and Backman, 1983
	Infiernillo Channel, Gulf of California, Mexico	Annual	–	–	–	6210.9–71187.0 (1997-2000, 2009,2010)	–	–	Lopez-Calderon et al., 2016
	Gulf of California, Mexico	Annual	–	–	–	17,000–30,000 (1995, 2010)	–	–	
Northwestern Atlantic	Chesapeake Bay USA	Perennial	–	–	72.2 ± 127.8	6,969–9,752	322.2 ± 388.9	–	Harwell and Orth, 2002
	Chesapeake Bay USA (FE)	Perennial	–	Sep-May	–	–	–	–	Orth and Moore, 1983
	Chesapeake Bay USA	Perennial	Feb-May	–	353	8127	–	–	Silberhorn et al., 1983
	Chesapeake Bay USA (FE)	Perennial	–	–	–	–	–	–	Orth et al., 1994
	Chesapeake Bay USA (FO)	Perennial	–	–	90–120	650–950	–	–	Harwell and Rhode, 2007
	Virginia, Chesapeake Bay, USA	Perennial	Mar-Jun (1979)	Oct-Apr	424 ± 170	–	0–11	–	Orth and Moore, 1986
	Newport River, USA	Annual	Feb-Jul (2008)	Oct–	603 ± 157 (Mar, 2008)	53,513 ± 7,365	1,243 ± 147 (Sep, 2008)	–	Jarvis, 2009; Jarvis and Moore, 2010
	Back Sound, USA	Perennial	Feb-Jul (2008)	Oct–	463 ± 224 (May, 2008)	36,000 ± 8,595	232 ± 77 (Oct, 2008)	–	
	Northwest Creek, New York, USA (FE)	–	–	Oct-Dec	–	–	–	–	Churchill, 1983
European Coast	Limfjorden, Danmark	Perennial	Mar-Aug	–	79 ± 20 (May, 1990)	8,100 ± 1,600	–	–	Olesen and Sand-Jensen, 1994
	Lake Grevelingen, Netherlands	Perennial	Jun-Nov	Spring	–	13,463–14,876 (1988)	104–320 (Spring,1989)	–	van Lent and Verschuure, 1994
	VeerseMeer, Netherlands	Annual	Jun-Nov	Spring	–	46,261 (1988)	864 (Spring,1989)	–	
	Zandkreek, Netherlands	Annual	Jun-Nov	Spring	–	26,764 (1988)	128 (Spring,1989)	–	
	Zandkreek, Netherlands	Annual	Jul-Oct	–	10	200	–	–	Hootsmans et al., 1987
	Zandkreek, Netherlands	Annual	–	Jan-May	184 (Mar, 1989)	–	1,120	–	Harrison, 1993
	Isles of Scilly UK	Annual	Jun-Oct	–	–	–	–	–	Potouroglou et al., 2014
	Thau lagoon, France	Perennial	Mar-Jun	–	40 (Mar, 1995)	–	–	–	Laugier et al., 1999

In contrast, the reproduction recruitment cycle of *Z. marina* in HQB was quite different from that in SLL. The four processes of *Z. marina* in HQB were as follows (Figure 11B): (1) seeds mainly germinated from October and the overwintering seedlings began clonal growth in April; (2) overwintering shoots began clonal growth in April; (3) both seedling shoots and overwintering shoots began rapid clonal growth at the beginning of June, proceeded to flowering in the middle of April and produced seeds from June to July, during which time mature seeds were dispersed into the sediment to form a seed bank; (4) flowering shoots withered after reproduction, while shoots that did not flower decayed with time and some of them overwintered, entering the next cycle.

### Plant reproductive effort

The reproduction of *Z. marina* has been well-described, indicating that the flowering shoot densities or ratios are highly variable along its geographic distribution (Table 5). Flowering shoots are the primary factor in sexual reproduction, and the flowering shoot density and percent in adult shoots, as well as potential seed yields, are all of importance to analyze eelgrass reproductive efforts. Reproductive strategies varied between the patch and center sites in SLL. Flowering shoots at the center site of SLL appeared from the beginning of May and disappeared at the end of July, while their appearance at the patch site occurred 1 month later. The appearance of flowering shoots varied by region. They were first observed in the middle of April in HQB, where the water temperature was higher than in SLL, and the seed release timing was also about 10 days earlier than in SLL. The flowering shoots were observed from June until October in Netherlands (51°33′N, 3°53′E; Harrison, 1993), where the mean air temperature (3–18.5°C; Vermaat et al., 1987) was lower than SLL and HQB. This indicated that temperature might be an important determinant of flowering and seed release, therefore, temperature might be an important factor affecting sexual reproduction. Flowering shoot density increased after their emergence but did not increase rapidly until June in both SLL and HQB. There was a significant difference between the two regions. Flowering shoot density peaked in June in SLL when the water temperature was 19°C, but they ebbed quickly as the seeds matured. The flowering shoot percent was 48.99 ± 29.79% in SLL, while the percent in the Chesapeake Bay, USA is lower, at 11–19% of the total density (Jarvis and Moore, 2010). The potential seed reproduction rates in the two regions were high compared with populations in Europe (Hootsmans et al., 1987; Harrison, 1993; Table 5).

Sexual reproduction (flowering shoots, seed production, and seed size and weight) are different between annual and perennial forms of *Z. marina* (Jarvis, 2009). The population of SLL is a mixture of the annual and perennial forms of *Z. marina*, while the HQB population has developed a perennial life history. The reproductive efforts of the *Z. marina* populations of SLL and HQB were site-specific. The population of SLL had a higher flowering density and seed production rate, but the seeds produced were lighter and smaller than those from HQB. The lower densities of flowering shoots found in HQB relative to SLL may be related to the different life-history model. It may also be related to HQB being open to the sea and, therefore, subjected to stronger currents and sediment movement, which may result in the breakage and transportation of the reproductive shoots. In addition, the number of spathes per flowering shoot of the *Z. marina* population of SLL (7–17 spathes per flowering shoot) was within the range (5–18 spathes per flowering shoot) reported for this species elsewhere (Phillips and Backman, 1983; Phillips et al., 1983; Silberhorn et al., 1983; Morita et al., 2007). However, the average number of spathes per flowering shoot of the HQB population (13–26 spathes per flowering shoot) was 21.5 ± 5.32, which is higher than in the aforementioned reports, and it may occur to compensate for the breakage and loss of the reproductive shoots. The number of spathes per flowering shoot is inversely related to water temperature (Silberhorn et al., 1983), and a higher number of spathes per flowering shoot has been reported for populations in Canada [36 spathes per flowering shoot; Keddy, 1987] and Northern France (20 spathes per flowering shoot; Jacobs and Pierson, 1981). In the present study, the number of spathes per flowering shoot of the HQB population was higher in a higher water temperature, which may be the result of adaptation to local conditions (e.g., currents and nutrient availability; Short, 1983; Jackson et al., 2017).

The difference in *Z. marina* seed sizes among populations has been documented (Wyllie-Echeverria and Ackerman, 2003). The larger and heavier seeds are produced under environments that are more favorable to adult plant survival and growth (Baskin and Baskin, 1998). For this study, *Z. marina* seeds from SLL were notably smaller in width and length, and lighter in weight compared with those from HQB. The weight of the *Z. marina* seeds from SLL (5.78 ± 0.34 mg) was lower than those of other populations (ca. 8 mg; Granger et al., 2003), including HQB seeds which had a weight of 8.81 ± 0.03 mg. The heavier seeds may contain a greater amount of resources for seed germination, seedling establishment and growth (Fenner and Thompson, 2005). In contrast, the smaller and lighter seeds of *Z. marina* are more easily dispersed (Symonides, 1988; Shipley et al., 1989; Rees, 1996; Jarvis, 2009), which can protect them from stressful environment and predation.

### Seed banks

The formation of an eelgrass seed bank is a crucial part of eelgrass sexual reproduction, and the seed bank is usually transient (Orth et al., 2006b). Our study demonstrated that viable seed densities at the end of seed germination approached zero in both regions. The seed bank size in SLL was relatively larger, with a peak densities of 552.21 ± 204.94 seeds m^−2^, compared with the size of the seed bank in the Chesapeake Bay, USA, which had a maximum densities of 91 ± 35 seeds m^−2^ (Jarvis and Moore, 2010). Compared with *Z. marina*, which has a reported range of 0–1,200 seeds m^−2^ in perennial meadows (Harrison, 1993; Harwell and Orth, 2002; Lee et al., 2007; Morita et al., 2007), the seed bank sizes in SLL and HQB were both in the middle of this range. Although seed bank size is related to the nature of the species and is usually under strong genetic control, microsites also play essential roles (Orth et al., 2006b), as do environmental factors. The seed bank densities in SLL peaked (552.21 ± 204.94 seeds m^−2^) in October after seed burial was accomplished. This was also true in HQB. A similar result was documented in the Netherlands, where the eelgrass seed densities reached its highest level in the fall or winter after the seed burial period. However, seed health decline dramatically affected seed bank size, with ~80% of viable seeds dying over the winter before the next year's germination period (Harrison, 1993). In any seed bank, the number of viable seeds decreases once seeds have germinated, since they are removed from the seed bank (Leck et al., 1989). In our present study, over 57.87% (December) of viable seeds were dying prior to the germination period. The number of viable seeds decreased with time due to germination, decay, predation, etc.; and seed coat density in sediment might reflect the loss of seed bank. The seed loss reported here may be due to damage to the seed coat, disease, predation, or parasitism (Keddy and Patriquin, 1978; Harrison, 1993; Fishman and Orth, 1996; Nakaoka, 2002; Manley et al., 2015). Jarvis (2009) found that time was the only factor that had a significantly negatively effect on seed viability in both North Carolina, USA and Virginia, USA seeds in the ambient seed bank. Here, we found that seed banks in SLL (patch and center sites) were generally very patchy (Fenner, 1995) even at very small spatial scales (cm to tens of cm; Inglis, 2000; Harwell and Orth, 2002). In addition, there was a significant difference in the size of the seed banks between the two sites we monitored in SLL, with the seed density of the center site being much higher than that of the patch site. The direct reason might be seed yield, because there was a much greater potential seed production owing to the higher flowering shoot density at the center site. Furman et al. (2015) found that seed dispersal varied systematically from 1.85 to 5.31 m for naked seeds, which ensured that the eelgrass seeds would mainly be maintained in vegetated areas.

### Seed germination and seedling establishment

Most studies reported germination during winter and spring (Orth and Moore, 1983; Phillips and Backman, 1983; Harrison, 1993; Orth et al., 1994, 2003; van Lent and Verschuure, 1994; Harwell and Orth, 1999; Olesen, 1999; Bintz and Nixon, 2001; van Katwijk and Wijgergangs, 2004; Greve et al., 2005). However, most seeds germinated during autumn for some *Z. marina* populations elsewhere (Churchill, 1983; Orth and Moore, 1983; Phillips and Backman, 1983; Moore et al., 1993; Orth et al., 1994, 2003; Plus et al., 2003). The common factors that affect germination are usually both internal, such as genetic control, and external, such as water temperature, which was very important in the field (Orth et al., 2000). Temperature is an important factor affecting the timing for germination of *Z. marina* seeds, and studies reported that 6–11°C (Probert and Brenchley, 1999) or 10–15°C (Abe et al., 2008) was the optimal temperature range for germination. Seed germination mainly began in the middle of March in SLL, when seedling density was very low, and seedling density peaked in April, and ended in late June, when no new seedlings germinated. However, the situation in the Netherlands was a little different with an earlier beginning in late January and an earlier ending in May. Additionally, seed germination approached 50% in the same year and there was still 10% of seeds left to geminate the next year (Harrison, 1993).

Densities of seedlings were highly correlated with seed bank size at the two sites of SLL. Flowering shoots and seed bank at the center site were higher than those at the patch site. However, the SRC at the patch site (50.52%) was higher than that at the center site (41.36%). It was might due to a relatively fast clonal growth at the patch site (Figure 8). The seedling density peaked in April in the center site, with a mean density of 296.88 ± 274.27 seedlings m^−2^. With the collective efforts of germination and growth, seedling density increased from the middle of March and peaked in April, when the water temperature in SLL ranged from 7.4 to 6.0°C. Seedlings that emerged in Zandkreek, northwestern Netherlands in February but did not peak until April, declined from May and then seed germination ended after June (Harrison, 1993). Although it is in the North Temperate Zone with a lower water temperature, the seed germination period was much longer than in SLL. The seedling density declined after the peak, mainly resulted from seedling mortality (Harrison, 1993), due to the exhaustion of seed nutrients and the failure in new resources competition (Orth et al., 2006b). In SLL, seed germination ended in May with no new germinated seedlings and a higher water temperature above 21.8°C.

Seedlings that established and grew gradually begin to branch using rhizomes. Our study suggested that seedling branching in SLL commenced in the beginning of May, and a similar result was documented in the Netherlands (Harrison, 1993). With the growth of seedlings and increasing of water temperature, eelgrass shoots from seedlings flowered in the same year in SLL, as did other adult shoots. The same phenomenon was not observed in the Chesapeake Bay, USA (Jarvis and Moore, 2010).

Eelgrass reproduction in SLL was more prolific relative to other populations because of the higher flowering shoot rate. *Z. marina* is common along the coast of the northern temperate provinces of China. den Hartog and Yang (1990) reported that the southernmost locality is Shijiusuo, Rizhao City, Shandong Province, but ecological datum on the species in this area are limited. However, *Z. marina* was not found at this locality with unpolluted water conditions during a recent survey on seagrass habitats in the coastal waters of China. The aforementioned survey has also indicated that the southernmost distribution limit of *Z. marina* has been moving northward from Shijiusuo (35°21′N, 119°32′E) to Qingdao (36°03′N, 120°20′E), which might be caused by the temperature increase associated with global warming. In Rizhao, a gradual warming trend (0.248°C/10a) has been observed in the past 50 years (Lu et al., 2012). In the present study, we observed that *Z. marina* seed germination in the field occurred in the autumn for the HQB population when the temperature fell below 20°C. This was consistent with our laboratory germination experiment that showed a high proportion of HQB seeds germinated within 5 weeks at the optimal germination temperature (15°C). A gradual warming trend (0.281°C/10a) has been documented in the past 50 years, especially for the lowest temperature increase in winter seasons [0.338°C/10a; Liu et al., 2014]. Although the latitude of SLL is higher than that of HQB by just 1°, the temperature of SLL changes rapidly and is much lower than that of HQB in winter seasons. The intertidal zone of HQB have little chance to be covered by ice, while most of the intertidal zone of SLL is covered by ice in the winter and in some years the whole lagoon is covered by ice. Thus, *Z. marina* seeds from the HQB population, with a greater seed size, were acclimated to an autumn germination with seedlings easily surviving the warmer winter. By contrast, the population in SLL was not acclimated to an autumn germination, but a spring germination instead, when the seedling had many months to grow and establish before a harsh winter. Moreover, the population of *Z. marina* cannot complete their sexual reproductive cycle prior to low temperature onset and ice formation. Phillips et al. (1983) reported that ice in the winter is an important cause of mortality at the northern margin of the range in the Bering Sea, and Churchill (1983) found that *Z. marina* seedling growth and development occurred during the autumn and spring, but not in the winter. Thus, wintertime conditions might prevent seeds from germinating in autumn. Cabaco and Santos (2009) reported that a longer period between seed dispersal and germination could allow for large-scale resilience by adapting to a wider range of environmental conditions. In our germination experiment, we observed that the germination period of *Z. marina* seeds from SLL was longer than that of seeds from HQB, and the maximum GV(D) of the SLL population was just 0.76 ± 0.14. Thus, the SLL population, having a smaller seed size, may need a longer period of dormancy to overwinter, and eventually acclimated to a spring germination.

Studies conducted in the laboratory and field worldwide have shown that seagrasses respond to disturbances of various origins (anthropogenic or natural) and types (extreme temperatures, eutrophication and hydrodynamic stress) by changing their sexual reproductive efforts (Cabaco and Santos, 2012; Jarvis et al., 2012). This alteration may be partly reflected in the seed size variation of eelgrass (Harper, 1977; Wyllie-Echeverria and Ackerman, 2003; Fenner and Thompson, 2005). The seagrass ecosystem of SLL is protected from strong currents and waves from Rongcheng Bay by a narrow and long sandbar east of the lake, while the seagrass ecosystem in HQB, which is an open bay to the Yellow Sea, is influenced by strong currents and waves. In the present study, we observed that the seed size and wet weight of the *Z. marina* population of HQB are significantly greater than those of SLL (*P* < 0.05). The large morphological inter-population variations in *Z. marina* seeds may result from adaptations to local specific environments (e.g., currents and waves) in which plants develop. We hypothesized that heavy and large seeds are resistant to strong currents and waves, which allow them to remain close to the parent plant and avoid seed loss, while smaller seeds are easily dispersed close to the parent plant in more peaceful current and wave areas.

Based on our investigations in large coastal areas of China, the northern location of eelgrass is Changshan Island (Changhai County) in Dalian City, Liaoning Province (39°18′N, 122°35′E; Figure 1), and the island waters are influenced by cool wintertime conditions, and strong currents and waves. The seed size (93.26 ± 20.35 mm^3^) and wet weight (11.68 ± 0.41 mg) of the Changshan Island population is significantly greater than those of SLL and HQB (*P* < 0.05), and seed germination also occurred in the spring (March-May; Table 5). Thus, morphological and biological variations in eelgrass seeds may be a result of adaptations to specific locations. The temperature regime may control morphological and phonological variations.

In summary, this study made a comparison in the characteristics of sexual reproduction in *Z. marina* at Swan Lake and Huiquan Bay, and the role of sexual reproduction in the population recruitment of these two populations. Swan Lake and Huiquan Bay populations differed in timing of the reproductive phase (flowering, seed release, and seed germination), potential seed production, seed parameters (weight and size), and seedling density, resulting in differences in plant reproductive effort and sexual contribution to population recruitment. The germination percent and germination value were also different between the two populations in laboratory germination experiment. Eelgrass seeds were dispersed heterogeneously, and densities of seeds showed a clear temporal pattern with substantial variances. Our work indicated that temperature regime may induce shifts in sexual recruitment strategies. We suggest that further detailed work be conducted on effects of gradual warming on the temperate species *Z. marina*, including distribution and sexual reproduction and recruitment.

## Author contributions

ScX: conceived and designed the laboratory seed germination experiment, performed the experiments, analyzed, and interpreted the data, contributed reagents, materials, analysis tools, wrote the paper, prepared figures and tables, reviewed drafts of the paper and approved of the submitted and final versions. PW: conceived and designed the field experiment, performed the experiment, analyzed the data, contributed reagents, materials, analysis tools, wrote the paper, prepared figures, and approved of the submitted and final versions. YZ: conceived and designed all experiments, interpreted the data, revised and reviewed drafts of the paper, and approved of the submitted and final versions. XZ, RG, XL, BL, XS, SX, and SY: acquired the data, revised the paper, and approved of the submitted and final versions.

### Conflict of interest statement

The authors declare that the research was conducted in the absence of any commercial or financial relationships that could be construed as a potential conflict of interest.
